# Association between the triglyceride-glucose index and the risk of left ventricular aneurysm formation among patients with acute ST-segment elevation myocardial infarction

**DOI:** 10.3389/fcvm.2025.1677922

**Published:** 2025-10-30

**Authors:** Dong Hu, Jing Zhao, Tin Huang, Qinshuo Zhao, Man-Hua Chen

**Affiliations:** ^1^ Department of Cardiology, The Central Hospital of Wuhan, Tongji Medical College, Huazhong University of Science and Technology, Wuhan, China; ^2^ Key Laboratory for Molecular Diagnosis of Hubei Province, The Central Hospital of Wuhan, Tongji Medical College, Huazhong University of Science and Technology, Wuhan, China; ^3^ Hubei Provincial Engineering Research Center of Intestinal Microecological Diagnosis and Treatment Technology and Clinical Application, Wuhan, Hubei, China; ^4^ Department of Cardiology, Renmin Hospital of Wuhan University, Wuhan, China

**Keywords:** triglyceride-glucose index, left ventricular aneurysm, acute ST-segment elevation myocardial infarction, primary percutaneous coronary intervention, risk prediction

## Abstract

**Introduction:**

The triglyceride–glucose (TyG) index has been closely associated with a range of cardiovascular diseases. However, the association between the TyG index and left ventricular aneurysm (LVA) formation in patients with acute ST-segment elevation myocardial infarction (STEMI) who underwent primary percutaneous coronary intervention (PCI) remains unknown.

**Methods:**

This study recruited 551 patients in the first cohort and 471 patients in the validation cohort. To determine the independent risk factors associated with LVA formation, a multivariable logistic regression analysis was conducted. The predictive capability of both the TyG index and the composite variable for LVA formation was evaluated through the use of ROC curve analysis.

**Results:**

The prevalence of LVA was found to be 14.5% in the first cohort and 13.6% in the validation cohort. In both cohorts, a higher TyG index correlated with an increased incidence of LVA (20.5% vs. 8.0%, *P* < 0.001 in the first cohort; 18.4% vs. 8.6%, *P* = 0.002 in the validation cohort). The TyG index was consistently elevated in the LVA group in comparison to those without LVA (9.3 ± 0.7 vs. 8.9 ± 0.9, *P* < 0.001 in the first cohort; 9.4 ± 0.7 vs. 9.0 ± 0.7, *P* < 0.001 in the validation cohort). Analysis using multivariable logistic regression showed that an independent relationship exists between the TyG index and the risk of LVA in both the first (OR = 4.2, *P* = 0.004) and validation (OR = 2.11, *P* = 0.008) cohorts. The applicability of the TyG index in predicting LVA had C statistics of 0.736 for the first cohort and 0.738 for the validation cohort, which surpassed those of triglycerides and fasting plasma glucose. The composite variable consisting of the TyG index, left ventricular ejection fraction, and left anterior descending artery (LAD) as the culprit vessel significantly improved the discriminant power (C statistic = 0.913 in the first cohort and 0.89 in the validation cohort).

**Discussion:**

A higher TyG index was independently linked to the development of LVA in patients with acute STEMI who received primary PCI.

## Background

Left ventricular aneurysm (LVA) represents one of the most severe complications following acute myocardial infarction (AMI), characterized by the outward bulging of the infarcted myocardium during both systole and diastole (1). Among patients experiencing AMI, the incidence of LVA has been reported to range from 5%–15% (2). Furthermore, the occurrence of major adverse cardiovascular and cerebrovascular events is 2.5 times higher in patients with LVA compared to those without (3), primarily due to an increased incidence of arrhythmias, thromboembolic events, congestive heart failure, and cardiac rupture (3, 4). Given the significant incidence and mortality associated with LVA, it is crucial to identify AMI patients at elevated risk for LVA formation to facilitate early prevention and intervention strategies.

The triglyceride-glucose (TyG) index, derived from fasting triglyceride (TG) and blood glucose levels, has been validated as a reliable surrogate marker for insulin resistance, exhibiting high concordance with the hyperinsulinemic-euglycemic clamp (5, 6). Recent studies have increasingly demonstrated an association between the TyG index and cardiovascular diseases (CVDs). In a cohort study of 1,516 patients with symptomatic coronary artery disease, Li et al. reported a significant positive correlation between the TyG index and both coronary lesions and carotid plaques (7). Similarly, a multicenter, retrospective cohort study of 830 nondiabetic patients who underwent coronary artery bypass grafting identified an association between the TyG index and adverse cardiovascular events (6). Furthermore, a retrospective study involving 6,697 patients with chronic heart failure found a significant association between the TyG index and mortality risk (8). A systematic review by Tao et al. suggested that the metabolic fexibility, endothelial dysfunction, coagulation disorders, and smooth muscle cell dysfunction are likely the molecular mechanisms underlying the relationship between the TyG index and CVDs (9). However, the relationship between the TyG index and the risk of LVA development in patients experiencing acute STEMI emains inadequately elucidated. To address this knowledge gap, we conducted the present study to evaluate the predictive capacity of the TyG index regarding the risk of LVA formation in individuals experiencing acute ST-segment elevation myocardial infarction (STEMI) within the Han Chinese population.

## Methods

### Study design and participants

A total of 706 consecutive patients with acute STEMI who underwent primary percutaneous coronary intervention (PCI) between December 2018 and February 2023 in the Central Hospital of Wuhan were included in our study. Acute STEMI was diagnosed according to the fourth universal definition of myocardial infarction (10), which includes the following criteria: typical chest pain lasting over 30 min, with new ST-segment elevation at the J point in at least two contiguous leads of >2 mm (0.2 mV) in men or >1.5 mm (0.15 mV) in women on admission electrocardiogram, and an increase in cardiac enzyme levels above the 99th percentile cut-off point for cardiac troponin I (cTnI). The criteria for exclusion included cases of non-ischemic cardiomyopathy (such as hypertrophic and dilated types), congenital heart defects, ongoing infections, kidney or liver failure, malignant neoplasms, individuals with a life expectancy of less than one year, those who received thrombolytic therapy before hospital admission, and patients lost to follow-up. Finally, the association analysis included a total of 551 patients.

Additionally, a replicated cohort consisting of 619 patients diagnosed with acute STEMI, who had received primary PCI at Renmin Hospital of Wuhan University between July 2018 and June 2022, was enrolled consecutively to independently assess the predictive value of the TyG index regarding the formation of LVA. The criteria for exclusion included cases of non-ischemic cardiomyopathy (*N* = 11), congenital heart defects (*N* = 2), ongoing infections (*N* = 12), kidney or liver failure (*N* = 49), malignant neoplasms (*N* = 2), those who received thrombolytic therapy before hospital admission (*N* = 7), and patients lost to follow-up (*N* = 65). Finally, the association analysis included a total of 471 patients.

This study was conducted in accordance with the principles of the Declaration of Helsinki and received approval from the Review Board of the Central Hospital of Wuhan (WHZXKYL2023-091) and Renmin Hospital of Wuhan University (2023K-K007). All patients provided informed consent before participating.

### Echocardiography and PCI procedure

All patients underwent two-dimensional transthoracic echocardiography (TTE) upon admission and again at the conclusion of the first and sixth months of the follow-up period. The diagnosis of LVA was established through TTE based on the protocol delineated in the Coronary Artery Surgery Study (CASS) (11). The criteria for diagnosing LVA were as followings: (I) bulging of the left ventricular wall during both diastole and systole, exhibiting either akinesia or dyskinesia; (II) clear demarcation of the infarcted segment; and (III) absence of trabeculation in the affected segment.

Prior to primary PCI, all patients received aspirin (300 mg loading dose followed by 100 mg daily), clopidogrel (600 mg loading dose followed by 75 mg daily), or ticagrelor (180 mg loading dose followed by 90 mg twice daily). Additionally, a bolus of unfractionated heparin (UFH) was intravenously administered at a dose of 70 U/kg of body weight. The primary PCI procedure was performed using either the standard radial or femoral approach, in accordance with current guidelines. All patients have been deployed stent for the culprit artery. During the PCI procedure, the operator could decide the use of balloon pre-dilatation or post-dilatation, the type of stents (bare metal or drug-eluting), and the use of thrombus aspiration. In accordance with the operator judgment, the glycoprotein IIb/IIIa receptor inhibitor tirofiban was initiated during the PCI procedure with 10 μg/kg intracoronary bolus followed by 0.15 μg/kg/min intravenous infusion. Residual stenosis <10% in the culprit lesion after the procedure was regarded as technically successful stent implantation. The results of coronary angiograms, the extent and degree of stenosis of each major coronary artery vessel were analyzed and determined by two independent experts. Upon discharge, medical therapy was prescribed based on the patient's individual condition and guideline recommendations for secondary prevention.

### Data collection and definitions

Trained physicians blinded to the study's purpose were responsible for collecting patient demographic and clinical data from the electronic medical recording system, including age, gender, hypertension, diabetes, smoking status, left ventricular ejection fraction (LVEF), angiographic evaluation results, and medication used at discharge. Hypertension was defined as either receiving treatment for hypertension before admission or having a blood pressure measurement exceeding 140/90 mmHg. Diabetes mellitus was defined as a fasting plasma glucose level greater than 7.0 mmol/L, a postprandial blood glucose level greater than 11.1 mmol/L, a hemoglobin A1c level greater than 6.5%, or receiving treatment for diabetes mellitus. Smoking history was defined as having smoked more than 2 pack-years and/or having smoked within the past year. Fasting elbow venous blood samples were collected from all patients upon admission to the emergency room, between 7:00 and 9:00 a.m. To determine the peak value of cardiac enzyme levels, blood samples for troponin I (TnI) and lactate dehydrogenase (LDH) were obtained every 12 h during the first 48 h and every 24 h thereafter, after admission to the intensive coronary care unit, from a peripheral vein. The Gensini score was calculated using the method developed by Celebi et al. (12). The TyG index was calculated using the following formula: ln (fasting triglyceride (mg/dl) × FPG (mg/dl)/2) (9).

### Statistical analysis

Continuous variables were represented as mean ± standard deviation or as median with interquartile range, based on the normality of the data distribution. Categorical variables were presented as numbers (percent). The best cut-off value of the TyG index to stratify patients into two groups was determined to be 8.93 according to the receiver operating characteristic (ROC) curve analysis in the first cohort (Sensitivity: 0.638; Specificity: 0.73, Youden's Index: 0.368). Differences among groups were assessed using the chi-squared test for categorical variables, independent-sample t-test for continuous variables with a normal distribution, and Mann–Whitney *U* test for continuous parameters with skewed distribution. Univariate logistic regression analysis was utilized to examine the association between different variables and the risk of LVA formation. Variables with a significance level of *P* < 0.05 in the univariate analysis were included in the multivariate logistic regression analysis.

All statistical analyses in the present study were performed using SPSS 26 (IBM Corporation Armonk, NY, USA). All comparisons were two-sided, and a significance level of *p* < 0.05 was considered statistically significant.

## Results

### Patient characteristics

**Figure 1:**
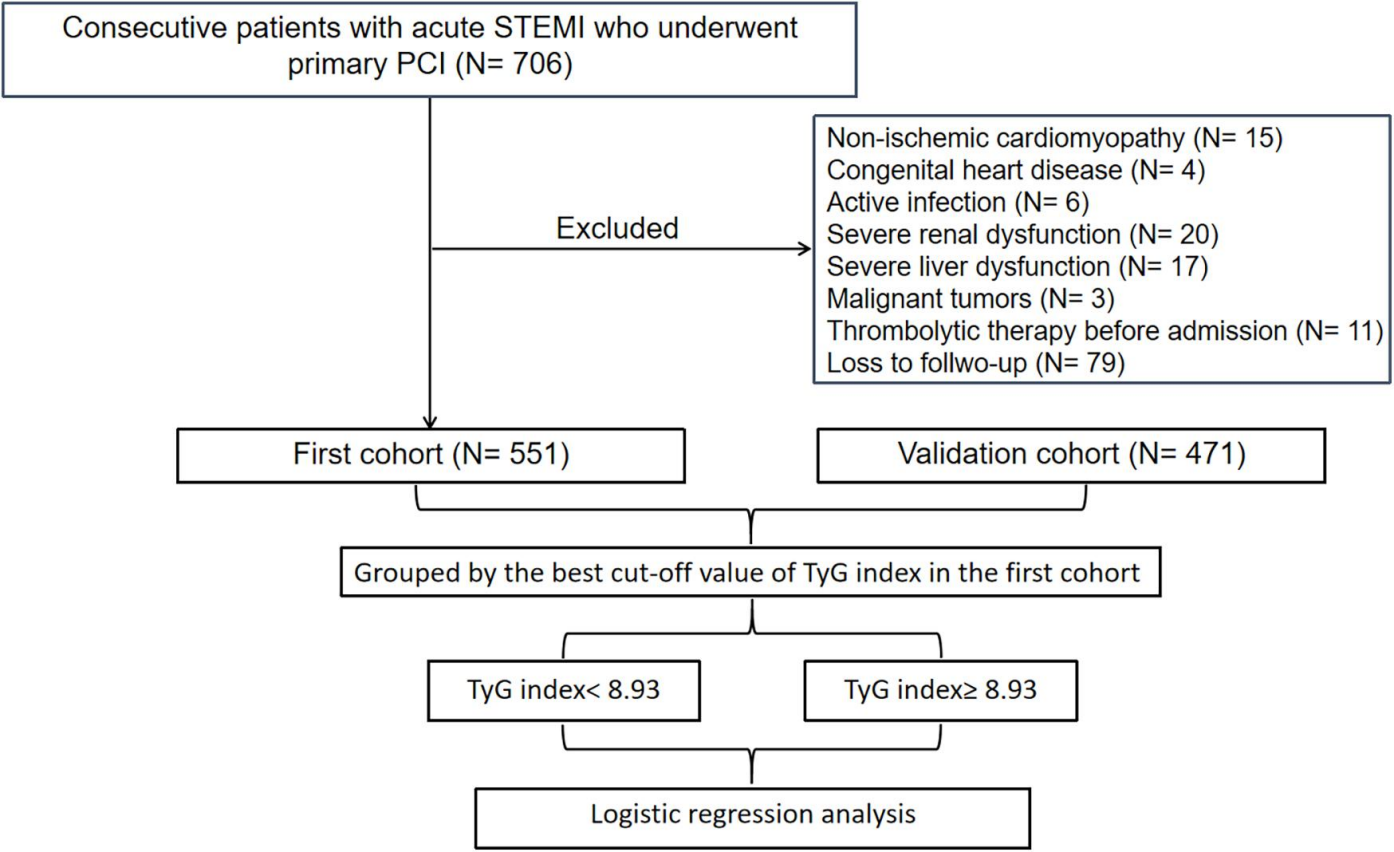
Flow diagram of patient selection. TyG, triglyceride-glucose; STEMI, acute ST-segment elevation myocardial infarction; PCI, percutaneous coronary intervention.

Figure 1 illustrates the comprehensive methodology workflow for this study. The baseline characteristics of the first cohort (*N* = 551) and the validation cohort (*N* = 471) are presented in Table 1. The average (± SD) age for the first cohort (19.8% female) and validation cohort (16.1% female) were 61.2 ± 12.9 and 60.5 ± 12.2 years, respectively. Over the follow-up duration for TTE, a total of 80 (14.5%) LVA in the first cohort and 64 (13.6%) in the validation cohort were observed.

**Table 1:** Baseline characteristics of the study population.

Variables	First cohort (*N* = 551)	Validation cohort (*N* = 471)	*P*-value
Demographics			
Gender, female, (%)	109 (19.8)	76 (16.1)	0.13
Age (years)	61.2 ± 12.9	60.5 ± 12.2	0.41
Medical history, *n* (%)			
Hypertension	300 (54.4)	260 (55.2)	0.81
Diabetes	176 (31.9)	138 (29.3)	0.36
Smoking	298 (54.1)	269 (57.1)	0.33
Laboratory parameters			
Sodium (mmol/L)	139.1 ± 3.1	139.1 ± 2.9	0.91
Potassium (mmol/L)	4.0 ± 0.5	4.1 ± 0.3	0.04
WBC (×109/L)	9.7 (7.8–12.1)	11.3 (9.5–13.8)	<0.001
RBC (×109/L)	4.5 ± 0.7	4.6 ± 0.6	0.00
Hemoglobin (g/L)	138.4 ± 21.4	140.7 ± 18.7	0.07
HbA1c, (%)	6.0 (5.6–6.9)	6.1 (5.7–7.4)	0.09
ALT, (U/L)	28.1 (17.9–48.4)	32.0 (17.0–53.0)	0.15
AST, (U/L)	58.5 (25–160.2)	71 (38–184)	<0.001
ALB, (g/L)	41.0 ± 5.3	39.1 ± 3.0	<0.001
LDH, (U/L)	312 (202–573)	458 (295–686)	<0.001
Uric acid (umol/L)	371.0 (307.8–439.3)	373.0 (311.8–446.0)	0.83
FPG (mmol/L)	6.7 (5.4–9.3)	6.7 (5.6–8.7)	0.72
TC (mmol/L)	4.7 ± 1.2	4.5 ± 1.0	0.00
TG (mmol/L)	1.4 (0.9–2.0)	1.4 (1.1–2.0)	0.06
HDL (mmol/L)	1.0 (0.8–1.1)	1.0 (0.8–1.2)	0.27
LDL (mmol/L)	3.1 ± 1.1	2.9 ± 1.0	0.01
C reactive protein (mg/L)	0.7 (0.2–1.9)	0.7 (0.2–2.0)	0.93
D-dimer (ug/ml)	0.4 (0.2–0.7)	0.4 (0.2–0.8)	0.33
Peak cTnI (ng/ml)	19.7 (3.5–50.0)	21.2 (7.0–39.3)	0.49
TyG index	9.0 ± 0.9	9.1 ± 0.7	0.03
LVEF, (%)	54.2 ± 8.5	49.0 ± 7.8	<0.001
SBP (mmHg)	129.3 ± 20.7	122.0 ± 20.1	<0.001
DBP (mmHg)	80.9 ± 14.4	76.2 ± 13.0	<0.001
Medication at hospital discharge, *n* (%)			
Statin	551 (100.0)	471 (100.0)	>0.99
Aspirin	551 (100.0)	471 (100.0)	>0.99
Clopidogrel/Ticagrelor	551 (100.0)	471 (100.0)	>0.99
Beta blocker	474 (86.0)	415 (88.1)	0.32
Spironolactone	198 (35.9)	160 (34.0)	0.51
ACEI/ARB	405 (73.5)	365 (77.5)	0.14
Thiazide/loop diuretic	199 (36.1)	162 (34.4)	0.57
Coronary artery injury			
LAD as Culprit vessel, (%)	289 (52.5)	283 (60.1)	0.01
Multiple vessel disease, (%)	376 (68.2)	306 (65.0)	0.27
Gensini Score	72 (47–92)	64 (40–83)	<0.001
LVA, (%)	80 (14.5)	64 (13.6)	0.60

WBC, white blood cell count; RBC, red blood cell count; HbA1c, glycated hemoglobin; ALT, alanine aminotransferase; AST, aspartate aminotransferase; ALB, albumin; LDH, lactate dehydrogenase; FPG, fasting plasma glucose; TC, total cholesterol; TG, triglyceride; HDL; high-density lipoprotein; LDL, low-density lipoprotein; TyG, triglyceride-glucose; LVEF, left ventricular ejection fraction; SBP, systolic pressure; DBP, diastolic pressure; ACEI/ARB, angiotensin converting enzym inhibitor/angiotensin receptor blocker; LAD, left anterior descending artery; LVA, left ventricular aneurysm.

Subsequently, we compared the baseline demographics and clinical characteristics of the patients in groups with and without LVA formation (Table 2). In the first cohort, individuals in the LVA group were older and displayed elevated levels of LDH, FPG, C-reactive protein, D-dimer, cTnI, TyG index, diastolic blood pressure (DBP), and gensini score. Additionally, there was a higher percentage of those using spironolactone, ACEI/ARB, and thiazide/Loop diuretics, with left anterior descending (LAD) artery noted as the culprit vessel when compared to individuals in the non-LVA group (*P* < 0.05). Conversely, the LVA group demonstrated a lower percentage of smokers and reduced levels of albumin and left ventricular ejection fraction (LVEF) relative to the non-LVA group (*P* < 0.05). In the validation cohort, patients in the LVA group were more likely to be female, exhibited higher levels of white blood cell count (WBC), LDH, FPG, C reactive protein, D-dimer, Peak cTnI, TyG index, gensini score, and a higher proportion of spironolactone use, ACEI/ARB use, thiazide/loop diuretic use, and LAD as culprit vessel compared to those in the non-LVA group (*P* < 0.05). Conversely, the level of hemoglobin and LVEF were lower in patients with LVA compared to those without LVA (*P* < 0.05).

**Table 2:** Baseline characteristics of the LVA and Non-LVA groups.

Variables	First cohort			Validation cohort		
	Non-LVA patients (*N* = 471)	LVA patients (*N* = 80)	*P*-value	Non-LVA patients (*N* = 407)	LVA patients (*N* = 64)	*P*-value
Demographics						
Gender, female, (%)	87 (18.5)	22 (27.5)	0.061	59 (14.5)	17 (26.6)	0.015
Age (years)	60.5 ± 12.8	65.1 ± 12.9	0.003	60.1 ± 12.2	63.1 ± 12.1	0.067
Medical history, *n* (%)						
Hypertension	256 (54.3)	44 (55)	0.93	229 (56.3)	31 (48.4)	0.242
Diabetes	147 (31.2)	29 (36.3)	0.378	116 (28.5)	22 (34.4)	0.337
Smoking	263 (55.8)	35 (43.8)	0.043	237 (58.2)	32 (50.0)	0.216
Laboratory parameters						
Sodium (mmol/L)	139.2 ± 3.0	138.8 ± 3.5	0.36	139.1 ± 2.9	139.4 ± 3.1	0.387
Potassium (mmol/L)	4.0 ± 0.5	4.0 ± 0.5	0.63	4.1 ± 0.3	4.1 ± 0.3	0.686
WBC (×109/L)	9.7 (7.8–12.1)	9.6 (7.6–12.5)	0.72	11.0 (9.4–13.8)	12.7 (10.4–14.5)	0.005
RBC (×109/L)	4.5 ± 0.7	4.4 ± 0.6	0.5	4.6 ± 0.6	4.4 ± 0.8	0.05
Hemoglobin (g/L)	139.1 ± 21.6	134.6 ± 20.0	0.08	141.9 ± 17.8	133.4 ± 22.9	0.006
HbA1c, (%)	6.0 (5.6–6.9)	6.1 (5.7–7.8)	0.18	6.1 (5.7–7.4)	6.1 (5.6–8.3)	0.915
ALT, (U/L)	27.8 (18.0–47.0)	31.3 (17.6–54.5)	0.47	32 (16–53)	33 (20.3–59.8)	0.201
AST, (U/L)	57.7 (25.0–154.7)	68.2 (26.1–233.2)	0.15	71 (39–179.5)	71 (35–248.5)	0.935
ALB, (g/L)	41.4 ± 5.2	38.9 ± 5.5	<0.001	39.2 ± 3.0	38.6 ± 2.6	0.102
LDH, (U/L)	284 (199–535)	471 (238–748)	<0.001	444 (280–657)	654 (394–968)	<0.001
Uric acid (umol/L)	373 (308–440)	352 (306–426)	0.32	370.0 (310.5–437.3)	400.5 (312.5–477.5)	0.102
FPG (mmol/L)	6.4 (5.2–8.9)	7.9 (6.7–12.5)	<0.001	6.6 (5.5–8.5)	7.7 (6.0–10.7)	0.008
TC (mmol/L)	4.7 ± 1.2	4.8 ± 1.1	0.26	4.5 ± 1.0	4.5 ± 1.14	0.68
TG (mmol/L)	1.3 (0.8–2.1)	1.6 (1.1–2.0)	0.079	1.3 (1.0–1.8)	1.5 (1.1–2.3)	0.086
HDL (mmol/L)	1.0 (0.8–1.1)	1.0 (0.8–1.2)	0.6	1.0 (0.8–1.2)	0.9 (0.8–1.2)	0.269
LDL (mmol/L)	3.1 ± 1.1	3.3 ± 1.1	0.11	2.9 ± 1.0	2.9 ± 1.0	0.891
C reactive protein (mg/L)	0.6 (0.2–1.6)	2.0 (0.4–4.3)	<0.001	0.6 (0.2–1.8)	1.9 (0.4–5.4)	<0.001
D-dimer (ug/ml)	0.4 (0.2–0.6)	0.5 (0.3–1.0)	0.002	0.4 (0.2–0.7)	0.8 (0.4–2.2)	<0.001
Peak cTnI (ng/ml)	16.2 (3.5–48.3)	26.8 (4.9–50.0)	0.04	19.5 (6.5–39.7)	32 (11.6–39.0)	<0.001
TyG index	8.9 ± 0.9	9.3 ± 0.7	<0.001	9.0 ± 0.7	9.4 ± 0.7	<0.001
LVEF, (%)	56.0 ± 7.1	43.2 ± 8.1	<0.001	50.5 ± 6.8	39.8 ± 6.6	<0.001
SBP (mmHg)	129.2 ± 21.0	129.6 ± 18.5	0.891	122.7 ± 20.0	117.4 ± 20.4	0.051
DBP (mmHg)	80.4 ± 14.7	84.1 ± 11.7	0.034	76.6 ± 12.7	73.9 ± 14.1	0.127
Medication at hospital discharge, *n* (%)						
Statin	471 (100.0)	80 (100.0)	>0.999	407 (100.0)	64 (100.0)	>0.999
Aspirin	471 (100.0)	80 (100.0)	>0.999	407 (100.0)	64 (100.0)	>0.999
Clopidogrel/Ticagrelor	471 (100.0)	80 (100.0)	>0.999	407 (100.0)	64 (100.0)	>0.999
Beta blocker	407 (86.4)	67 (83.8)	0.526	362 (88.9)	53 (82.8)	0.159
Spironolactone	143 (30.4)	55 (68.8)	<0.001	114 (28.0)	46 (71.9)	<0.001
ACEI/ARB	336 (71.3)	69 (86.3)	0.005	308 (75.7)	57 (89.1)	0.017
Thiazide/loop diuretic	145 (30.8)	54 (67.5)	<0.001	114 (28.0)	48 (75.0)	<0.001
Coronary artery injury						
LAD as Culprit vessel, (%)	221 (46.9)	68 (85.3)	<0.001	224 (55.0)	59 (92.2)	<0.001
Multiple vessel disease, (%)	315 (66.9)	61 (76.3)	0.096	267 (65.6)	39 (60.9)	0.467
Gensini Score	65 (44–89)	88 (64–106)	<0.001	60 (40–80)	80 (48–96)	<0.001

WBC, white blood cell count; RBC, red blood cell count; HbA1c, glycated hemoglobin; ALT, alanine aminotransferase; AST, aspartate aminotransferase; ALB, albumin; LDH, lactate dehydrogenase; FPG, fasting plasma glucose; TC, total cholesterol; TG, triglyceride; HDL; high-density lipoprotein; LDL, low-density lipoprotein; TyG, triglyceride-glucose; LVEF, left ventricular ejection fraction; SBP, systolic pressure; DBP, diastolic pressure; ACEI/ARB, angiotensin converting enzym inhibitor/angiotensin receptor blocker; LAD, left anterior descending artery; LVA, left ventricular aneurysm.

According to the criterion of Maximum Youden Index, we determined the optimal cut-off value for the TyG index to stratify patients into two groups: TyG < 8.93 and TyG ≥ 8.93. In the first cohort (Table 3), patients in the TyG ≥ 8.93 group were younger and exhibited higher levels of red blood cell count (RBC), hemoglobin, glycated hemoglobin (HbA1c), alanine aminotransferase (ALT), FPG, total cholesterol (TC), TG, low-density lipoprotein (LDL), C reactive protein, systolic pressure (SBP), DBP, as well as a higher prevalence of diabetes and LVA formation. On the contrary, patients with TyG ≥ 8.93 had lower levels of potassium, high-density lipoprotein (HDL), and LVEF. In the validation cohort (Table 4), the differences observed between the TyG < 8.93 and TyG ≥ 8.93 groups were comparable to those in the first cohort, with the exception of age, potassium, hemoglobin, LVEF, and SBP, which showed no significant difference. Besides, the levels of WBC and uric acid increased, while the proportion of multiple vessel disease decreased in the TyG ≥ 8.93 group.

**Table 3:** Baseline characteristics of patients stratified by TyG index in the first cohort.

Variables	TyG < 8.93 (*N* = 263)	TyG ≥ 8.93 (*N* = 288)	*P*-value
Demographics			
Gender, female, (%)	48 (18.3)	61 (21.2)	0.39
Age (years)	62.7 ± 12.1	59.8 ± 13.4	0.01
Medical historyy, *n* (%)			
Hypertension	136 (51.7)	164 (56.9)	0.22
Diabetes	45 (17.1)	131 (45.5)	<0.001
Smoking	139 (52.9)	159 (55.2)	0.58
Laboratory parameters			
Sodium (mmol/L)	4.0 ± 0.5	4.0 ± 0.5	0.807
Potassium (mmol/L)	139.6 ± 2.8	138.7 ± 3.3	<0.001
WBC (×109/L)	9.7 (7.7–12.0)	9.8 (7.8–12.2)	0.59
RBC (×109/L)	4.4 ± 0.6	4.5 ± 0.7	0.022
Hemoglobin (g/L)	136.0 ± 19.1	140.6 ± 23.1	0.011
HbA1c, (%)	5.8 (5.5–6.2)	6.4 (5.8–8.3)	<0.001
ALT, (U/L)	25.6 (15.9–43.8)	31.2 (18.9–51.4)	0.03
AST, (U/L)	61.8 (24.4–169.9)	56.9 (25.9–156.5)	0.5
ALB, (g/L)	40.9 ± 5.0	41.2 ± 5.6	0.579
LDH, (U/L)	311 (200–585)	316 (205–556)	0.94
Uric acid (umol/L)	359 (304–433)	377 (311–445)	0.35
FPG (mmol/L)	5.6 (4.9–6.9)	8.0 (6.3–11.7)	<0.001
TC (mmol/L)	4.4 ± 1.2	4.9 ± 1.1	<0.001
TG (mmol/L)	0.9 (0.6–1.3)	2.0 (1.6–2.9)	<0.001
HDL (mmol/L)	1.0 (0.9–1.2)	0.9 (0.8–1.1)	<0.001
LDL (mmol/L)	3.0 ± 1.1	3.2 ± 1.1	0.003
C reactive protein (mg/L)	0.6 (0.2–1.6)	0.8 (0.3–2.2)	0.046
D-dimer (ug/ml)	0.4 (0.2–0.7)	0.4 (0.2–0.7)	0.82
Peak cTnI (ng/ml)	19.7 (3.9–50.0)	19.8 (3.1–50.0)	0.43
TyG index	8.3 ± 0.6	9.6 ± 0.6	<0.001
LVEF, (%)	55.0 ± 8.3	53.4 ± 8.6	0.033
SBP (mmHg)	126.5 ± 18.8	131.8 ± 22.0	0.003
DBP (mmHg)	78.7 ± 13.5	83.0 ± 14.8	<0.001
Medication at hospital discharge, *n* (%)			
Statin	263 (100.0)	288 (100.0)	>0.99
Aspirin	263 (100.0)	288 (100.0)	>0.99
Clopidogrel/Ticagrelor	263 (100.0)	288 (100.0)	>0.99
Beta blocker	232 (88.2)	242 (84.0)	0.16
Spironolactone	86 (32.7)	112 (38.9)	0.13
ACEI/ARB	185 (70.3)	220 (76.4)	0.11
Thiazide/loop diuretic	86 (32.7)	113 (39.2)	0.11
Coronary artery injury			
LAD as Culprit vessel, (%)	132 (50.2)	157 (54.5)	0.31
Multiple vessel disease, (%)	175 (66.5)	201 (69.8)	0.41
Gensini Score	71.5 (46–92)	74 (48–92)	0.91
LVA, (%)	21 (8.0)	59 (20.5)	<0.001

WBC, white blood cell count; RBC, red blood cell count; HbA1c, glycated hemoglobin; ALT, alanine aminotransferase; AST, aspartate aminotransferase; ALB, albumin; LDH, lactate dehydrogenase; FPG, fasting plasma glucose; TC, total cholesterol; TG, triglyceride; HDL; high-density lipoprotein; LDL, low-density lipoprotein; TyG, triglyceride-glucose; LVEF, left ventricular ejection fraction; SBP, systolic pressure; DBP, diastolic pressure; ACEI/ARB, angiotensin converting enzyme inhibitor/angiotensin receptor blocker; LAD, left anterior descending artery; LVA, left ventricular aneurysm.

**Table 4:** Baseline characteristics of patients stratified by TyG index in the validation cohort.

Variables	TyG < 8.93 (*N* = 232)	TyG ≥ 8.93 (*N* = 239)	*P*-value
Demographics			
Gender, female, (%)	37 (15.9)	39 (16.3)	0.913
Age (years)	61.6 ± 11.9	59.4 ± 12.4	0.054
Medical history, *n* (%)			
Hypertension	123 (53.0)	137 (57.3)	0.348
Diabetes	45 (19.4)	93 (38.9)	<0.001
Smoking	123 (53.0)	146 (61.1)	0.077
Laboratory parameters			
Sodium (mmol/L)	4.1 ± 0.3	4.1 ± 0.3	0.19
Potassium (mmol/L)	139.3 ± 2.7	138.9 ± 3.2	0.097
WBC (×109/L)	10.8 (9.2–13.2)	11.8 (9.8–14.2)	0.008
RBC (×109/L)	4.5 ± 0.6	4.7 ± 0.6	0.009
Hemoglobin (g/L)	139.4 ± 17.6	142.1 ± 19.8	0.12
HbA1c, (%)	5.8 (5.5–6.2)	6.8 (5.9–8.5)	<0.001
ALT, (U/L)	29.5 (15.3–48.8)	34.0 (19.0–59.0)	0.038
AST, (U/L)	71 (36.3–186.3)	70.5 (39–178.3)	0.712
ALB, (g/L)	39.0 ± 1.9	39.3 ± 3.7	0.216
LDH, (U/L)	448 (273.5–662.5)	471.5 (320–703.3)	0.242
Uric acid (umol/L)	357 (299.3–423.8)	386 (329–461.3)	0.002
FPG (mmol/L)	5.8 (5.2–6.7)	8.5 (6.6–12.2)	<0.001
TC (mmol/L)	4.2 ± 0.9	4.8 ± 1.1	<0.001
TG (mmol/L)	1.1 (1.0–1.3)	1.9 (1.5–2.4)	<0.001
HDL (mmol/L)	1.1 (0.9–1.2)	0.9 (0.8–1.1)	<0.001
LDL (mmol/L)	2.8 ± 0.8	3.1 ± 1.0	0.002
C reactive protein (mg/L)	0.5 (0.2–1.8)	1.0 (0.3–2.5)	<0.001
D-dimer (ug/ml)	0.4 (0.2–0.7)	0.4 (0.2–1.0)	0.177
Peak cTnI (ng/ml)	23.3 (6.5–40.9)	19.8 (7.6–38.6)	0.806
TyG index	8.6 ± 0.2	9.6 ± 0.6	<0.001
LVEF, (%)	49.4 ± 7.2	48.6 ± 8.3	0.316
SBP (mmHg)	121.0 ± 19.3	123.0 ± 20.9	0.277
DBP (mmHg)	75.0 ± 12.4	77.4 ± 13.4	0.048
Medication at hospital discharge, *n* (%)			
Statin	232 (100.0)	239 (100.0)	>0.99
Aspirin	232 (100.0)	239 (100.0)	>0.99
Clopidogrel/Ticagrelor	232 (100.0)	239 (100.0)	>0.99
Beta blocker	206 (88.8)	209 (87.4)	0.652
Spironolactone	69 (29.7)	91 (38.1)	0.056
ACEI/ARB	173 (74.6)	192 (80.3)	0.134
Thiazide/loop diuretic	71 (30.6)	91 (38.1)	0.088
Coronary artery injure			
LAD as Culprit vessel, (%)	138 (59.5)	145 (60.7)	0.793
Multiple vessel disease, (%)	164 (70.7)	142 (59.4)	0.01
Gensini Score	60 (40–80)	72 (40–84)	0.244
LVA, (%)	20 (8.6)	44 (18.4)	0.002

WBC, white blood cell count; RBC, red blood cell count; HbA1c, glycated hemoglobin; ALT, alanine aminotransferase; AST, aspartate aminotransferase; ALB, albumin; LDH, lactate dehydrogenase; FPG, fasting plasma glucose; TC, total cholesterol; TG, triglyceride; HDL; high-density lipoprotein; LDL, low-density lipoprotein; TyG, triglyceride-glucose; LVEF, left ventricular ejection fraction; SBP, systolic pressure; DBP, diastolic pressure; ACEI/ARB, angiotensin converting enzym inhibitor/angiotensin receptor blocker; LAD, left anterior descending artery; LVA, left ventricular aneurysm.

### Tyg index and the incidence of LVA

**Figure 2:**
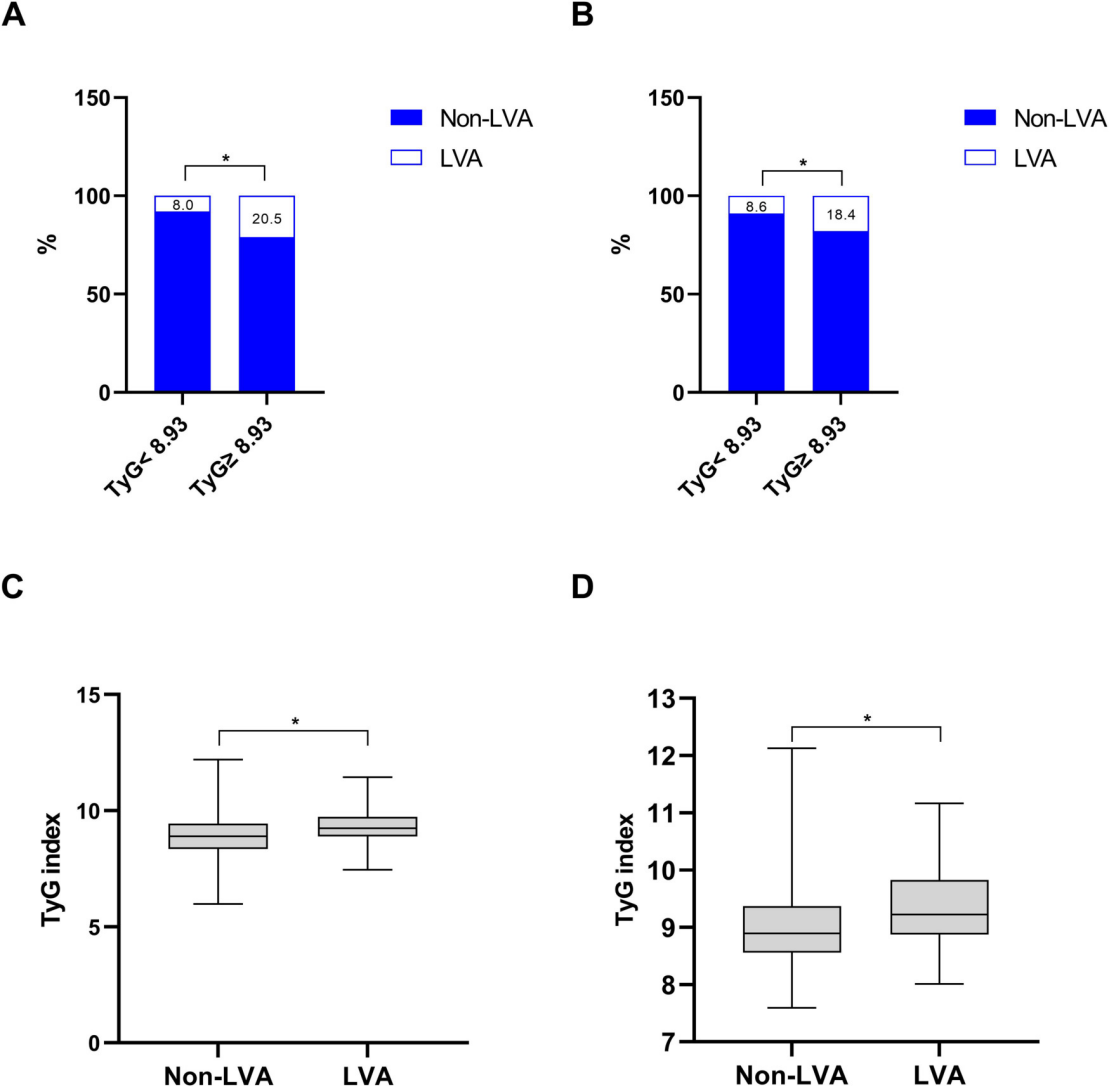
The association between the TyG index and the prevalence of LVA **(A,B)** and comparison of the TyG index level between the LVA and non-LVA groups **(C,D)** in the first **(A,C)** and validation **(B,D)** cohorts. TyG, triglyceride-glucose; LVA, left ventricular aneurysm; * *P* < 0.05.

As shown in Figures 2A,B, the incidence of LVA formation increased with the rise of TyG index (8.0% vs. 20.5%, *P* < 0.001 in the first cohort; 8.6% vs. 18.4%, *P* = 0.002 in the validation cohort). Furthermore, the LVA group exhibited a significantly higher TyG index than the non-LVA group (9.3 ± 0.7 vs. 8.9 ± 0.9, *P* < 0.001 in the first cohort; 9.4 ± 0.7 vs. 9.0 ± 0.7, *P* < 0.001 in the validation cohort; Figures 2C,D).

**Figure 3:**
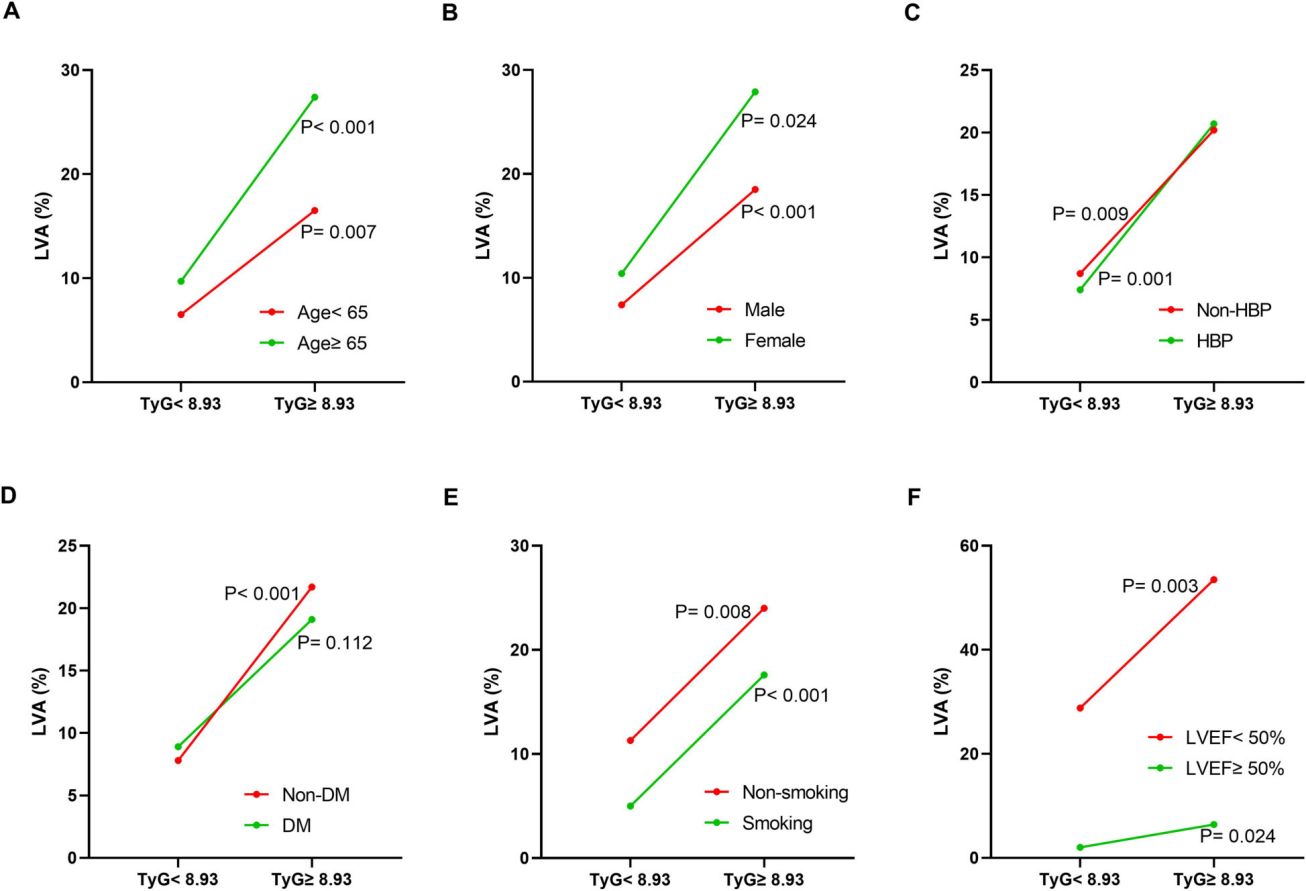
The impact of the TyG index on the prevalence of LVA across subgroups of age **(A)**, gender **(B)**, HBP status **(C)**, DM status **(D)**, smoking status **(E)**, and LVEF **(F)** in the first cohort. TyG, triglyceride-glucose; LVA, left ventricular aneurysm; HBP, hypertension; DM, diabetes mellitus; LVEF, left ventricular ejection fraction.

**Figure 4:**
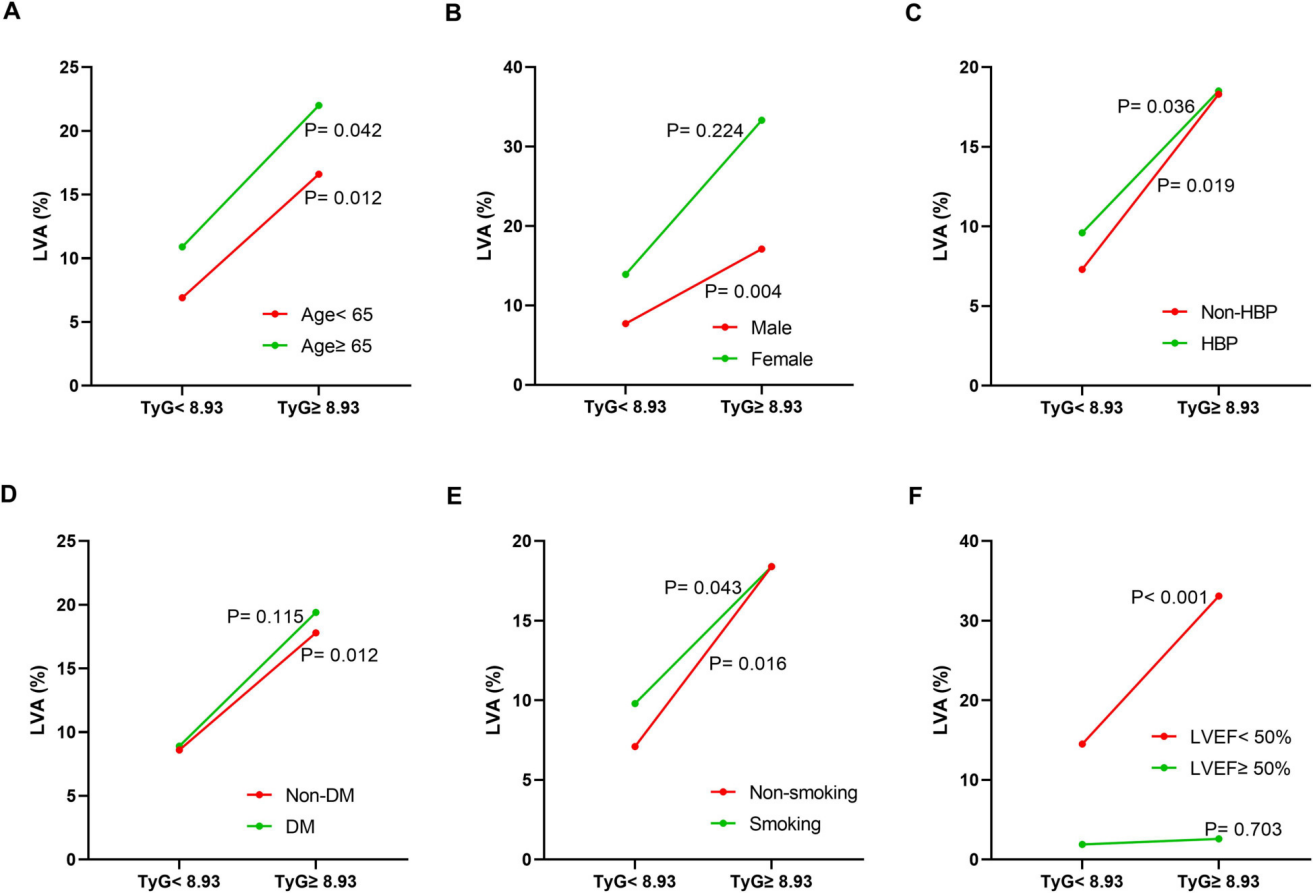
The impact of the TyG index on the prevalence of LVA across subgroups of age **(A)**, gender **(B)**, HBP status **(C)**, DM status **(D)**, smoking status **(E)**, and LVEF **(F)** in the validation cohort. TyG, triglyceride-glucose; LVA, left ventricular aneurysm; HBP, hypertension; DM, diabetes mellitus; LVEF, left ventricular ejection fraction.

Additionally, the relationship between the TyG index and LVA prevalence was evaluated across various subgroups. In the first cohort (Figure 3), as the TyG index increased, the prevalence of LVA rose in both male (7.4% vs. 18.5%, *P* < 0.001) and female (10.4% vs. 27.9%, *P* = 0.024), those aged < 65 (6.5% vs. 16.5%, *P* = 0.007) and ≥65 (9.7% vs. 27.4%, *P* < 0.001), individuals without hypertension (8.7% vs. 20.2%, *P* = 0.009) and with hypertension (7.4% vs. 20.7%, *P* = 0.001), non-diabetics (7.8% vs. 21.7%, *P* < 0.001), non-smokers (11.3% vs. 24%, *P* = 0.008) and smokers (5% vs. 17.6%, *P* < 0.001), as well as in patients with LVEF < 50% (28.8% vs. 53.5%, *P* = 0.003) and LVEF ≥ 50% (2% vs. 6.4%, *P* = 0.024). In the validation cohort (Figure 4), the similar results were observed as in the first cohort, except that there was no difference in the incidence of LVA in the subgroup of female (13.9% vs. 33.3%, *P* = 0.224) and LVEF ≥ 50% (1.9% vs. 2.6%, *P* = 0.703). Meanwhile, the TyG index between the non-LVA and LVA groups in these subgroups was also compared and the results were presented in Supplementary File 1 and Supplementary File 2.

### Association between the TyG index and other cardiometabolic risk factors

To investigate the relationship between the TyG index and other cardiometabolic risk factors, we conducted Spearman's rank or Pearson's correlation analysis. As shown in Table 5, the TyG index was positively associated with TC and C-reactive protein in both cohorts. Conversely, the TyG index showed a negative correlation with age and HDL.

**Table 5:** Association between TyG index and other cardiometabolic risk factors.

Variables	First cohort		Validation cohort	
	Correlation coefcient (r)	*P*-value	Correlation coefcient (r)	*P*-value
Age	−0.221	<0.001	−0.096	0.037
Uric acid	0.0447	0.298	0.188	<0.001
TC	0.222	<0.001	0.278	<0.001
HDL	−0.272	<0.001	−0.296	<0.001
LDL	0.076	0.077	0.09	0.051
C reactive protein	0.112	0.009	0.193	<0.001

TyG, triglyceride-glucose; TC, total cholesterol; HDL; high-density lipoprotein; LDL, low-density lipoprotein.

### Predictors for LVA formation

In order to evaluate the predictive value of variables for the risk of LVA formation in patients with acute STEMI patients who underwent PCI, logistic regression analysis was performed in the first cohort. As demonstrated in Table 6, a total of 18 variables were found to be associated with LVA formation in univariate logistic regression analysis. These variables included age, smoking, HbA1c, aspartate aminotransferase (AST), albumin (ALB), LDH, FPG, TG, C reactive protein, Peak cTnI, LVEF, DBP, spironolactone use, ACEI/ARB use, thiazide/loop diuretic use, LAD as culprit vessel, Gensini Score, and TyG index. The multivariable logistic regression model analysis were adjusted for age, smoking, HbA1c, AST, ALB, LDH, FPG, TG, C reactive protein, Peak cTnI, LVEF, DBP, spironolactone use, ACEI/ARB use, thiazide/loop diuretic use, LAD as culprit vessel, and Gensini Score. Table 6 revealed that only LVEF (OR = 0.82, 95% CI = 0.78–0.87, *P* < 0.001), ACEI/ARB use (OR = 3.19, 95% CI = 1.15–8.86, *P* = 0.026), LAD as culprit vessel (OR = 7.24, 95% CI = 2.79–18.75, *P* < 0.001) and TyG index (OR = 4.2, 95% CI = 1.60–11.06, *P* = 0.004) remained significantly correlated with the risk of LVA development.

**Table 6:** Association of TyG index with left ventricular aneurysm formation in logistic regression models in the first cohort.

Variables	Univariate logistic regression analysis			Multivariate logistic regression analysis		
	OR	95% CI	*P*-value	OR	95% CI	*P*-value
Age (years)	1.03	1.010–1.051	0.003			
Smoking	0.62	0.382–0.992	0.046			
HbA1c, (%)	1.17	1.030–1.324	0.015			
AST, (U/L)	1.00	1.000–1.003	0.024			
ALB, (g/L)	0.91	0.872–0.956	<0.001			
LDH, (U/L)	1.00	1.000–1.002	0.002			
FPG	1.10	1.05–1.16	<0.001			
TG (mmol/L)	0.77	0.61–0.97	0.029			
C reactive protein (mg/L)	1.10	1.039–1.154	0.001			
Peak cTnI (ng/ml)	1.01	1.003–1.023	0.012			
LVEF, (%)	0.83	0.805–0.865	<0.001	0.82	0.78–0.87	<0.001
DBP (mmHg)	1.02	1.001–1.034	0.036			
Spironolactone	5.05	3.024–8.420	<0.001			
ACEI/ARB	2.52	1.294–4.910	0.007	3.19	1.15–8.86	0.026
Thiazide/loop diuretic	4.67	2.812–7.754	<0.001			
LAD as Culprit vessel	6.41	3.381–12.155	<0.001	7.24	2.79–18.75	<0.001
Gensini Score	1.01	1.007–1.020	<0.001			
TyG index (<8.93/≥8.93)	2.97	1.75–5.04	<0.001	4.2	1.60–11.06	0.004

HbA1c, glycated hemoglobin; AST, aspartate aminotransferase; ALB, albumin; LDH, Lactate dehydrogenase; FPG, fasting plasma glucose; TG, triglyceride; LVEF, left ventricular ejection fraction; DBP, diastolic pressure; ACEI/ARB, angiotensin converting enzyme inhibitor/angiotensin receptor blocker; LAD, left anterior descending artery; LVA, left ventricular aneurysm; TyG, triglyceride-glucose.

Similarly, logistic regression analysis was performed in the validation cohort to investigate the relationship between various variables and the risk for LVA formation. Univariate logistic regression analysis identified 18 variables that were correlated with the risk for LVA formation, which included gender, WBC, RBC, hemoglobin, AST, ALB, LDH, FPG, TG, C reactive protein, Peak cTnI, LVEF, spironolactone use, ACEI/ARB use, thiazide/loop diuretic use, LAD as culprit vessel, Gensini Score, and the TyG index. Multivariate regression models corrected for variables included gender, WBC, RBC, hemoglobin, AST, ALB, LDH, FPG, TG, C reactive protein, Peak cTnI, LVEF, spironolactone use, ACEI/ARB use, thiazide/loop diuretic use, LAD as culprit vessel, Gensini Score. Table 7 revealed that only C reactive protein (OR = 1.178, 95% CI = 1.023–1.356, *P* = 0.023), Peak cTnI (OR = 1.066, 95% CI = 1.033–1.1, *P* < 0.001), LVEF (OR = 0.772, 95% CI = 0.709–0.841, *P* < 0.001), LAD as culprit vessel (OR = 7.712, 95% CI = 1.894–31.405, *P* = 0.004), and the TyG index (OR = 2.11, 95% CI = 1.21–3.66, *P* = 0.008) remained significantly correlated with the risk for LVA development using multivariate logistic regression analysis.

**Table 7:** Association of TyG index with left ventricular aneurysm formation in logistic regression models in the validation cohort.

Variables	Univariate logistic regression analysis			Multivariate logistic regression analysis		
	OR	95% CI	*P*-value	OR	95% CI	*P*-value
Gender	2.133	1.148–3.964	0.017			
WBC (×109/L)	1.109	1.039–1.183	0.002			
RBC (×109/L)	0.586	0.379–0.904	0.016			
Hemoglobin (g/L)	0.977	0.964–0.990	<0.001			
AST, (U/L)	1.002	1.001–1.003	<0.001			
ALB, (g/L)	0.88	0.77–0.99	0.047			
LDH, (U/L)	1.002	1.001–1.002	<0.001			
FPG (mmol/L)	1.05	1.001–1.10	0.045			
TG (mmol/L)	1.24	1.03–1.51	0.026			
C reactive protein (mg/L)	1.106	1.046–1.169	<0.001	1.178	1.023–1.356	0.023
Peak cTnI (ng/ml)	1.019	1.000–1.038	0.049	1.066	1.033–1.1	<0.001
LVEF, (%)	1.277	1.202–1.355	<0.001	0.772	0.709–0.841	<0.001
Spironolactone	0.152	0.085–0.274	<0.001			
ACEI/ARB	0.382	0.169–0.865	0.021			
Thiazide/loop diuretic	0.130	0.071–0.238	<0.001			
LAD as Culprit vessel	9.640	3.790–24.520	<0.001	7.712	1.894–31.405	0.004
Gensini Score	1.012	1.005–1.020	<0.001			
TyG index (<8.93/≥8.93)	2.780	1.607–4.810	<0.001	2.11	1.21–3.66	0.008

WBC, white blood cell count; RBC, red blood cell count; AST, aspartate aminotransferase; ALB, albumin; LDH, lactate dehydrogenase; FPG, fasting plasma glucose; TG, triglyceride; LVEF, left ventricular ejection fraction; ACEI/ARB, angiotensin converting enzyme inhibitor/angiotensin receptor blocker; LAD, left anterior descending artery; LVA, left ventricular aneurysm; TyG, triglyceride-glucose.

### Discriminative power analysis

**Figure 5:**
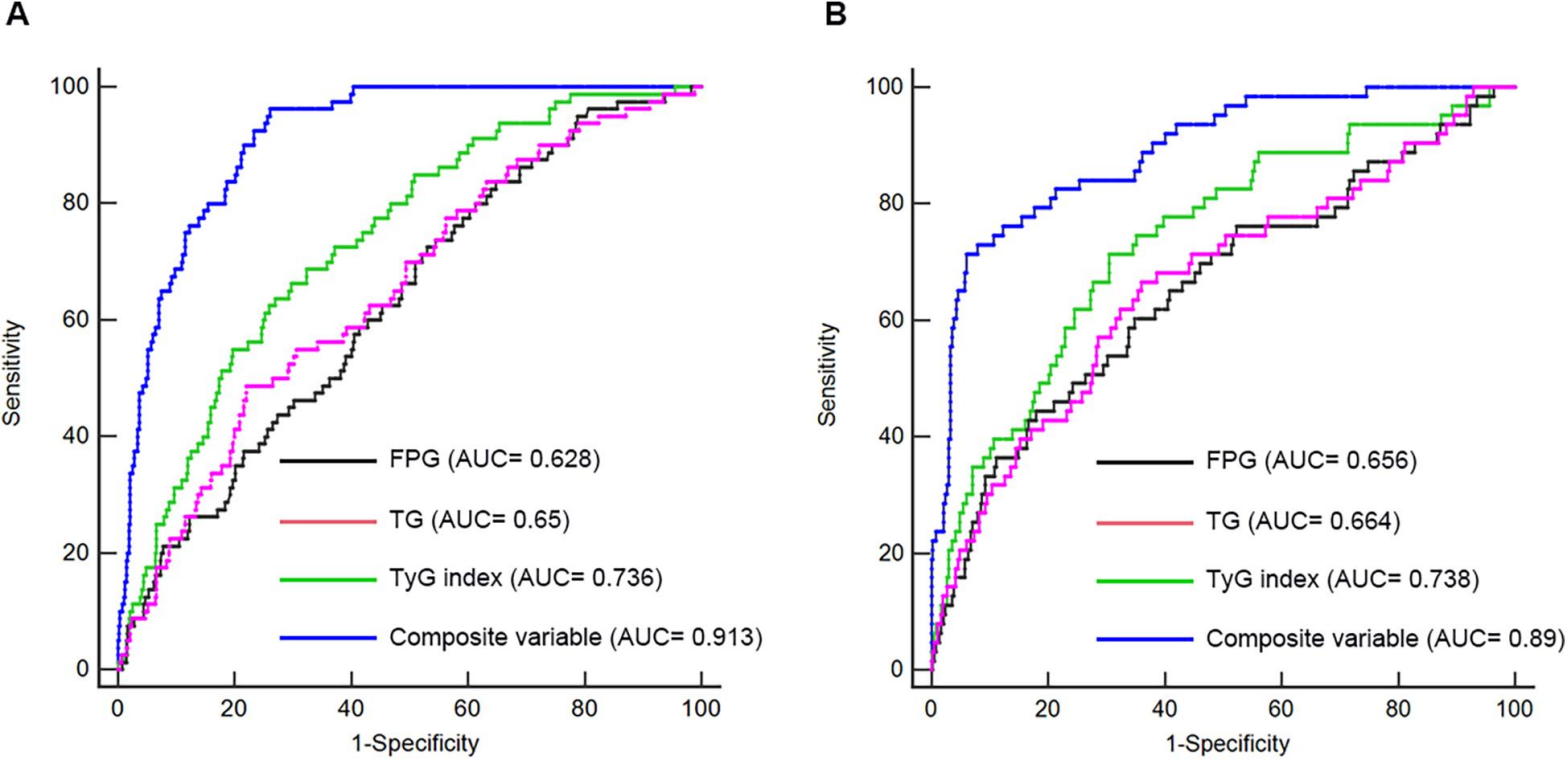
Receiver operating characteristic curve analysis of the TyG index to predict LVA formation in the first **(A)** and validation **(B)** cohorts. AUC, area under the curve; TyG, triglyceride-glucose; FPG, fasting plasma glucose; TG, triglyceride.

ROC analysis was conducted to evaluate and compare the predictive capabilities of FPG, TG, TyG index, and the composite variable (TyG index combined with LVEF and LAD as culprit vessel). In the first cohort, the average AUCs for FPG, TG, TyG index, and the composite variable were 0.628 (0.586–0.668), 0.65 (0.608–0.690), 0.736 (0.697–0.773), and 0.913 (0.886–0.935), respectively (Figure 5A and Table 8). In the validation cohort, the AUCs for FPG, TG, TyG index, and the composite variable were 0.656 (0.609–0.701), 0.664 (0.617–0.708), 0.738 (0.694–0.779), and 0.89 (0.856–0.918), respectively (Figure 5B and Table 8). In both cohorts, the TyG index demonstrated significantly higher AUCs for predicting the risk of LVA formation compared to TG (*P* < 0.05) and FPG (*P* < 0.05). Furthermore, the composite variable exhibited the highest predictive value (*P* < 0.001).

**Table 8:** Analysis of the ROC curve for predictive power of left ventricular aneurysm formation.

Cohorts	Variables	AUC	SE	95% CI
First cohort	FPG	0.628	0.032	0.586–0.668
	TG	0.65	0.033	0.608–0.690
	TyG index	0.736	0.028	0.697–0.773
	Composite variable	0.913	0.014	0.886–0.935
Validation cohort	FPG	0.656	0.04	0.609–0.701
	TG	0.664	0.04	0.617–0.708
	TyG index	0.738	0.035	0.694–0.779
	Composite variable	0.89	0.022	0.856–0.918

ROC, receiver operating characteristic; FPG, fasting plasma glucose; TG, triglyceride; TyG, triglyceride-glucose; AUC, area under curve; SE, standard error; CI, confidence interval.

### Subgroup analysis

**Figure 6:**
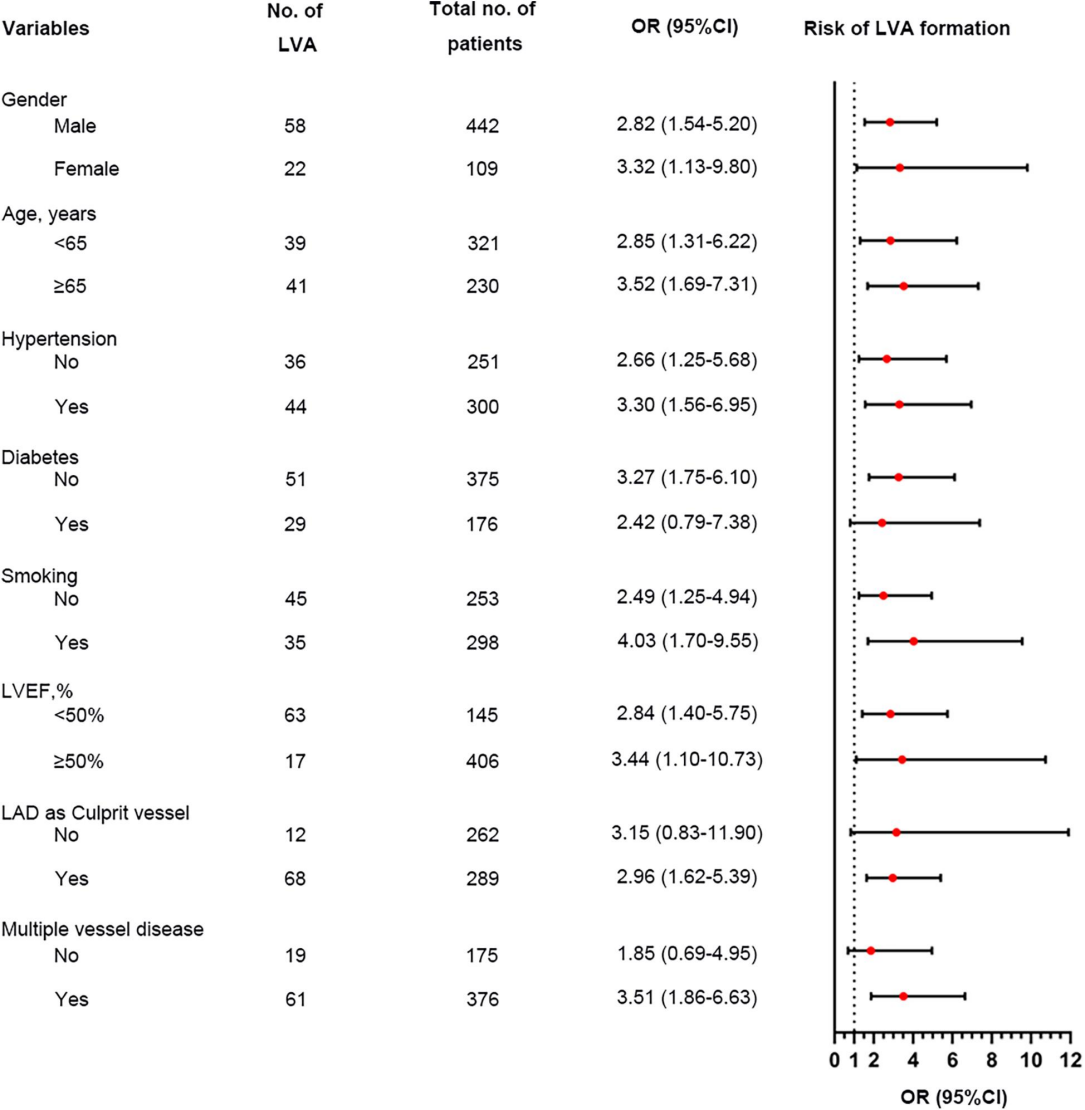
Forest plot investigating the association between the TyG index and the risk of LVA formation in different subgroups in the first cohort. TyG, triglyceride-glucose; LVA, left ventricular aneurysm; LVEF, left ventricular ejection fraction; LAD, left anterior descending artery; OR, odds ratio.

**Figure 7:**
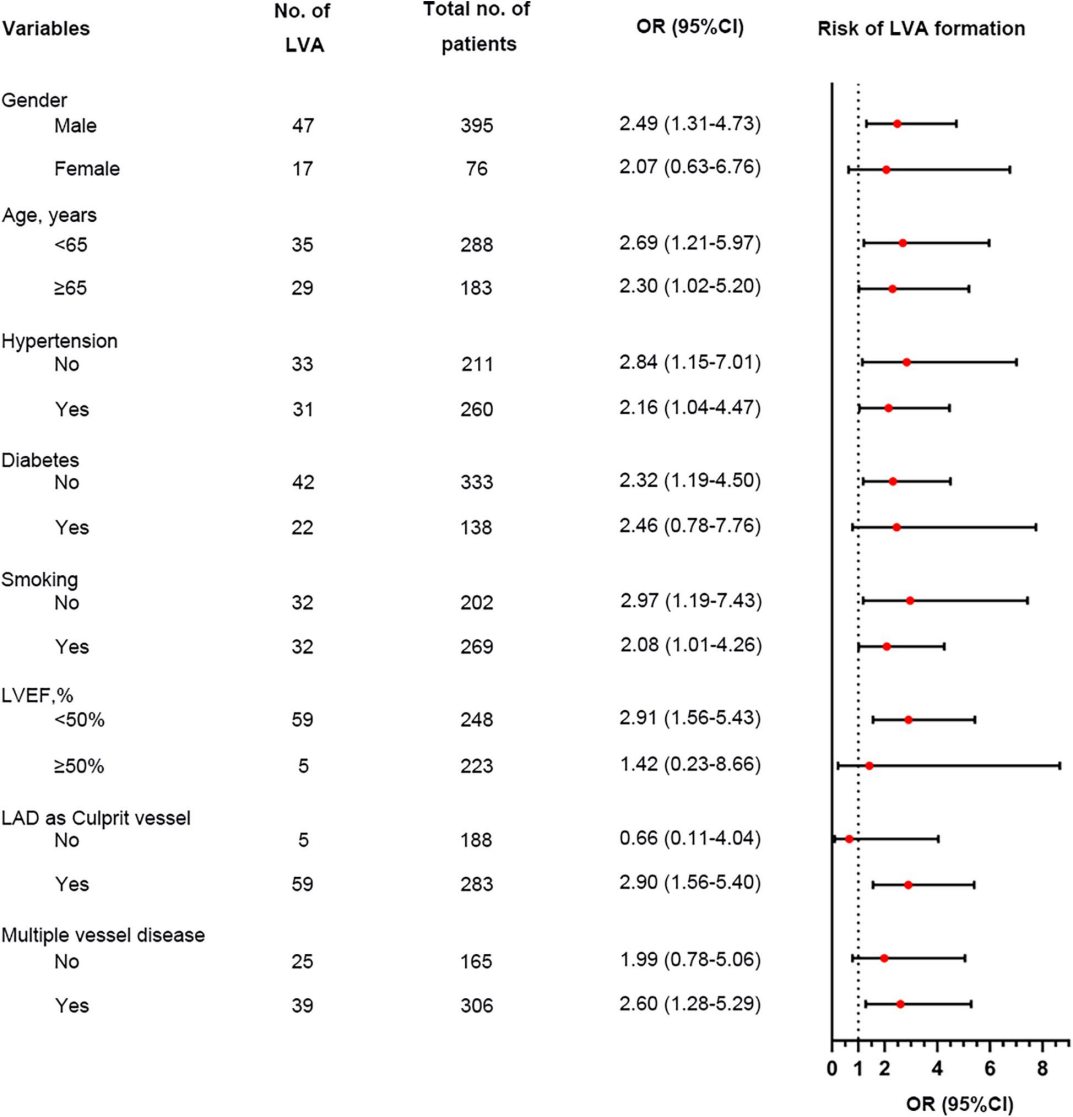
Forest plot investigating the association between the TyG index and the risk of LVA formation in different subgroups in the validation cohort. TyG, triglyceride-glucose; LVA, left ventricular aneurysm; LVEF, left ventricular ejection fraction; LAD, left anterior descending artery; OR, odds ratio.

We performed a subgroup analysis to further assess the independent predictive significance of the TyG index with regard to LVA formation across various clinically relevant subgroups. In the first cohort, the TyG index demonstrated a significant predictive effect on LVA formation in specific subgroups, including males and females, individuals with age <65 and ≥65 years, with and without hypertension, without diabetes, with and without smoking, individuals with LVEF < 50% and LVEF ≥ 50%, as well as individuals with LAD as the culprit vessel and those with multiple vessel disease (Figure 6). Notably, similar results were observed in the validation cohort, with the exception of the subgroups of females and individuals with LVEF ≥ 50% (Figure 7).

## Discussion

To the best of our knowledge, this study was the first to assess the association between the TyG index and the risk of LVA formation in patients with STEMI who underwent primary PCI. Our findings indicate a significant correlation between a higher TyG index and an increased risk of LVA formation in both the first and validation cohorts. The TyG index, serving as a surrogate marker of IR, showed a significant association with cardiometabolic risk factors. The relationship between the TyG index and LVA formation remained consistent across subgroups of male, individuals with age <65 and ≥65 years, with and without hypertension, without diabetes, with and without smoking, individuals with LVEF < 50%, as well as individuals with LAD as the culprit vessel and those with multiple vessel disease in both cohorts. Additionally, the TyG index displayed superior predictive ability for LVA formation compared to both TG and FPG. Notably, the composite variable exhibited the strongest predictive value.

Insulin resistance (IR) is a pathological condition marked by impairments in glucose uptake and oxidation, reduced glycogen synthesis, and diminished capacity to suppress lipid oxidation (13). Individuals with IR consistently exhibit elevated levels of circulating triglycerides (TG) and decreased concentrations of HDL (13). Recently, the triglyceride-glucose (TyG) index, derived from TG and fasting plasma glucose (FPG), has emerged as a novel surrogate marker for IR. A study conducted by Fernando et al. demonstrated a significant association between the TyG index and IR, indicating that it closely parallels the hyperinsulinaemic-euglycaemic clamp method in evaluating insulin sensitivity (14). Furthermore, the TyG index has been shown to outperform the HOMA2-IR in a Brazilian cohort with diverse adiposity and glucose tolerance levels (15). Additionally, the TyG index has been linked to various metabolic disorders, including diabetes and hypertension (16, 17). Consistent with previous studies (16, 18), our study also identified a higher likelihood of diabetes in individuals with an elevated TyG index. Moreover, our findings, corroborating previous studies (19, 20), also suggested that the TyG index was associated with other cardiometabolic risk factors.

Recently, a substantial of studies have been conducted to investigate the association between the TyG index and the cardiovascular diseases (21–25). A systematic review and meta-analysis by Amirmohammad et al. identified an association between the TyG index and both the prognosis and risk of heart failure (8). In a retrospective study involving 1,574 patients with acute coronary syndrome (ACS), Zhu et al. found that an elevated TyG index was positively correlated with in-stent restenosis following the implantation of drug-eluting stents (26). Furthermore, Zhang et al. conducted a study on 1,618 critically ill patients with coronary heart disease, demonstrating that the TyG index serves as a strong independent predictor of increased mortality (27). Recent findings also indicate that a higher TyG index can significantly predict future ischemic heart disease in nondiabetic Koreans (25). Nonetheless, no research has been performed to explore the relationship between the TyG index and the formation of LVA. This study provides the first evidence that TyG index ≥ 8.93 is significantly linked to a heightened risk of LVA formation when compared to TyG index < 8.93. Notably, the risk of LVA formation in patients with a TyG index ≥ 8.93 was more than 2-fold higher than in those with a TyG index < 8.93.

Recently, the LVA incidence after acute STEMI has decreased from 10%–30% to 8–15% due to the developments in the treatment (12). In accordance with these data, our study revealed that the occurrence of LVA in acute STEMI patients who underwent primary PCI were 14.5% in the first cohort and 13.6 in the validation cohort. Several predictors have been identified for LVA formation following acute myocardial infarction (AMI). You et al. reported that longer symptom-to-balloon time, higher initial and residual SYNTAX score, lower LVEF, and persistent ST segment elevation were independent predictors for LVA formation (3). A case–control study by Feng et al. identified decreased glomerular filtration rate and abnormal ferritin levels as the independent risk factors for LVA formation after AMI (28). In a retrospective study involving 1,823 STEMI patients, Zhang et al. found that female sex, peak NT-pro BNP, the time between the onset of pain and balloon time, presence of QS-waves on initial electrocardiogram were the independent predictors of early-onset LVA (29). However, there are still disagreements regarding these risk factors among different cohort studies. Furthermore, these studies primarily focused on single variables, most of which had modest or small effects on predicting LVA. In the present study, we introduced the TyG index for the first time as a predictor for LVA formation in patients with STEMI. The odds ratios for LVA occurrence in individuals with a TyG index of ≥8.93 were 4.2 in the first cohort and 2.11 in the validation cohort. Additionally, LVEF and LAD as culprit vessel were also identified as the independent predictor for LVA formation in both cohorts, which aligns with previous research (30, 31). Moreover, the TyG index showed enhanced predictive capability for LVA formation when compared to both TG and FPG, with the composite variable providing the most significant predictive value.

Importantly, no significant associations were identified between the TyG index and LVA in female patients, those with diabetes, individuals with an LVEF ≥ 50%, patients without LAD as the culprit vessel, and those without multivessel disease (*P* > 0.05) in the validation cohort. This lack of association is likely due to the limited sample size of female patients, those with diabetes, and individuals without multivessel disease, as well as the lower incidence of LVA in patients with an LVEF ≥ 50% and those without LAD as the culprit vessel.

The precise mechanisms underlying the relationship between the TyG index and LVA formation are not yet fully understood. First, the TyG index serves as a reliable indicator of insulin resistance. Previous studies have shown that insulin resistance may cause inflammation, oxidative stress, and apoptosis in cardiomyocytes (30, 31), which could increase the risk of LVA. Second, the association of an elevated TyG index with increased arterial stiffness and coronary artery calcification may represent another significant mechanism (32, 33).

Some limitations should be acknowledged in our present study. First, this study could not establish the causality between the TyG index and LVA formation due to the nature of an observational study. Second, routine measurements of insulin levels were not conducted in these patients, which hindered a meaningful comparison of the predictive values between HOMA-IR and the TyG index. Third, we concentrated exclusively on baseline levels of serum TG and FPG, neglecting the temporal variations in the TyG index that might have offered crucial insights into the underlying mechanisms. Forth, although our findings suggest a notable correlation between the TyG index and the risk of LVA formation, the practical clinical relevance of this correlation calls for validation in future prospective research. Finally, our findings are based on a cohort of patients from the Chinese Han population who experienced acute STEMI and underwent PCI treatment. The applicability of the proposed TyG index cut-off value (8.93) and the model to other ethnicities, patients with non-STEMI, or those treated with different strategies requires further validation.

## Conclusions

In conclusion, our research has shown that the TyG index serves as an important predictor for LVA formation in individuals with acute STEMI receiving primary PCI. Furthermore, the TyG index on its own demonstrated strong predictive capabilities for LVA development, while the combination of the TyG index, LVEF, and the LAD as the culprit vessel notably enhanced the ability to discriminate. Overall, our results indicate the promising application of the TyG index in clinical settings as a preferred predictor for LVA formation in patients with acute STEMI who have undergone primary PCI.

## Data Availability

The original contributions presented in the study are included in the article/Supplementary Material, further inquiries can be directed to the corresponding authors.

## Supplementary material

Supplementary File 1 Comparison of the TyG index between non-LVA and LVA groups in the subgroups of age (**A**), gender (**B**), HBP status (**C**), DM status (**D**), smoking status (**E**), and LVEF (**F**) in the first cohort. TyG, triglyceride-glucose; LVA, left ventricular aneurysm; HBP, hypertension; DM, diabetes mellitus; LVEF, left ventricular ejection fraction.

Supplementary File 2 Comparison of the TyG index between non-LVA and LVA groups in the subgroups of age (**A**), gender (**B**), HBP status (**C**), DM status (**D**), smoking status (**E**), and LVEF (**F**) in the validation cohort. TyG, triglyceride-glucose; LVA, left ventricular aneurysm; HBP, hypertension; DM, diabetes mellitus; LVEF, left ventricular ejection fraction.
