# Supplementary figures and images for: Association between the triglyceride-glucose index and the risk of left ventricular aneurysm formation among patients with acute ST-segment elevation myocardial infarction
Source: Front Cardiovasc Med. 2025 Oct 30;12:1677922. doi: 10.3389/fcvm.2025.1677922 (PMC12611940; doi:10.3389/fcvm.2025.1677922)

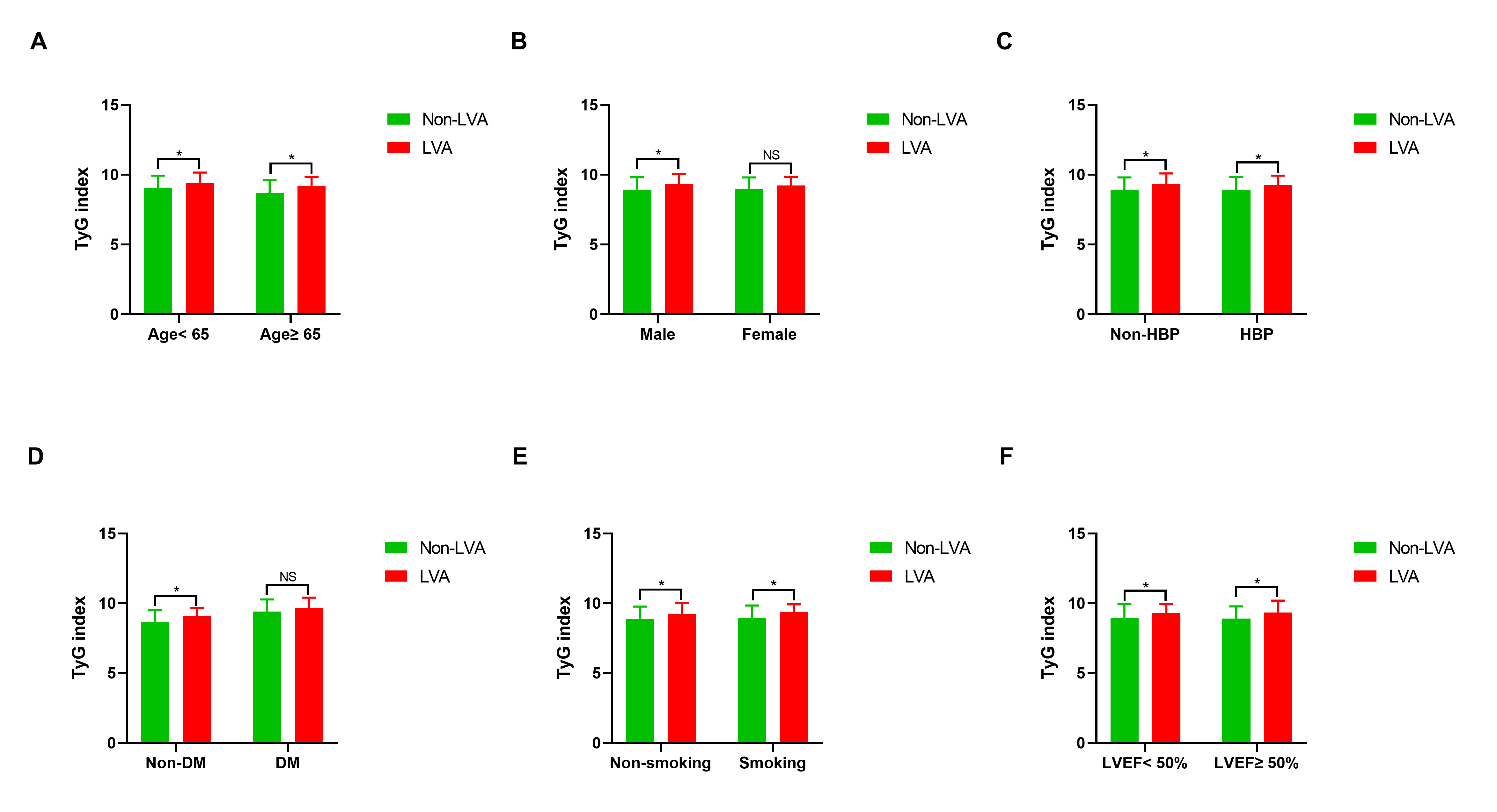

Supplement: Supplementary File 1 — Comparison of the TyG index between non-LVA and LVA groups in the subgroups of age (A), gender (B), HBP status (C), DM status (D), smoking status (E), and LVEF (F) in the first cohort. TyG, triglyceride-glucose; LVA, left ventricular aneurysm; HBP, hypertension; DM, diabetes mellitus; LVEF, left ventricular ejection fraction. [file Image1.tif]

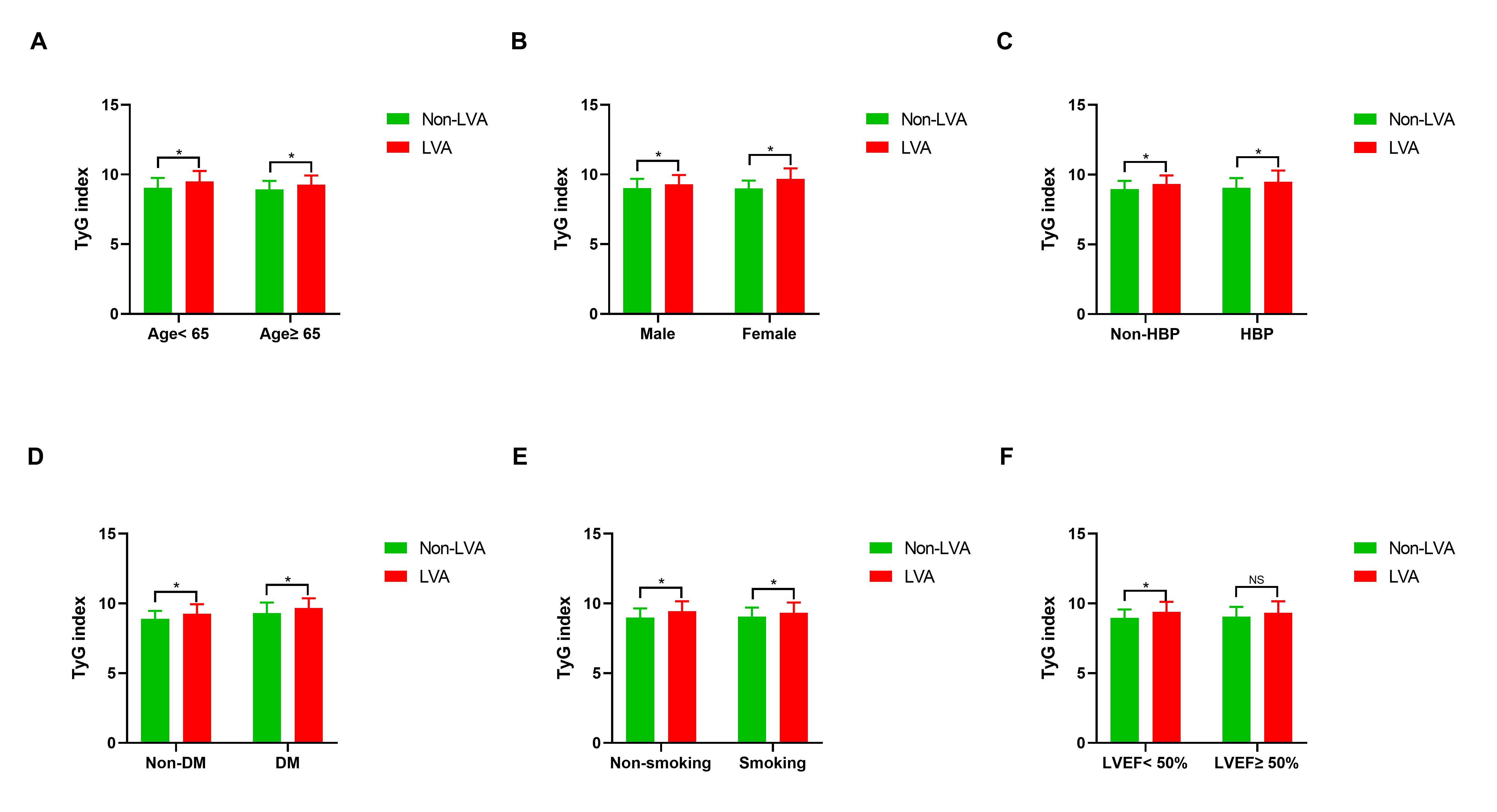

Supplement: Supplementary File 2 — Comparison of the TyG index between non-LVA and LVA groups in the subgroups of age (A), gender (B), HBP status (C), DM status (D), smoking status (E), and LVEF (F) in the validation cohort. TyG, triglyceride-glucose; LVA, left ventricular aneurysm; HBP, hypertension; DM, diabetes mellitus; LVEF, left ventricular ejection fraction. [file Image2.tif]
